# P-2161. Evaluating the Clinical Utility of Next-Generation Sequencing Cell-Free DNA Testing in Pediatric Patients: A Focus on Medical History

**DOI:** 10.1093/ofid/ofae631.2315

**Published:** 2025-01-29

**Authors:** Hussain Alkhafaji, Rachel Downey, Tyler Durham, Zuena Karim, Susan Russo, Sarah E McGwier, Alyssa Tough, Joanie Billeaud, Sarmistha Bhaduri Hauger

**Affiliations:** The University of Texas Southwestern Medical Center, Dallas, Texas; Dell Children's Medical Center of Central Texas, Austin, Texas; The University of Texas Southwestern Medical Center, Dallas, Texas; The University of Texas at Austin, Austin, Texas; Dell Children's Medical Center, Austin, TX; Dell Children’s Medical Group, Autsin, Texas; Dell Children's Medical Center of Central Texas, Austin, Texas; Dell Children's Medical Center of Central Texas, Austin, Texas; Dell Children's Medical Center; Dell Medical School at the University of Texas at Austin, Austin, Texas

## Abstract

**Background:**

The Karius test is a noninvasive, cell-free next-generation sequencing (NGS) test that can detect over 1,000 pathogens from a single blood sample. Validating the Karius test's useful clinical applications in pediatric patients is necessary to elucidate its benefits and limitations compared to conventional diagnostic testing. This pilot study aims to expand on existing evidence for Karius indications by investigating its utility among pediatric patients with specific underlying diagnoses.

**Figure 1:**
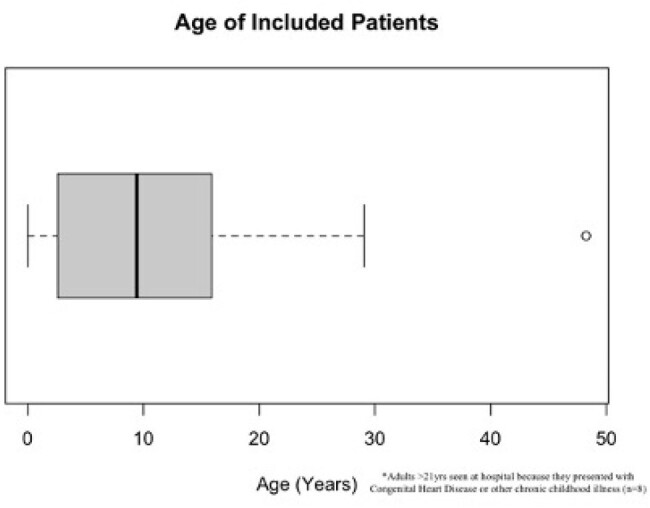
Age Distribution of Patients in Sample

**Methods:**

A retrospective chart review was conducted, consisting of 251 consecutive pediatric patients [03/2018-06/2022] (fig 1) for whom a Karius NGS test was performed at a free-standing 240-bed children’s hospital and affiliated clinic in Central Texas. Statistical analysis was conducted to evaluate whether a child’s underlying diagnosis was associated with clinically useful Karius results.

The Karius result was considered clinically useful if it was the first or only diagnostic test used to make the diagnosis, had negative predictive value essential to the plan of care, or contributed to changes in the antimicrobial regimen. Underlying diagnoses were categorized into ten groups (fig 2). Statistical analysis was conducted using R software via Pearson’s Chi-Squared Test and Fischer’s Exact Test.

**Figure 2:**
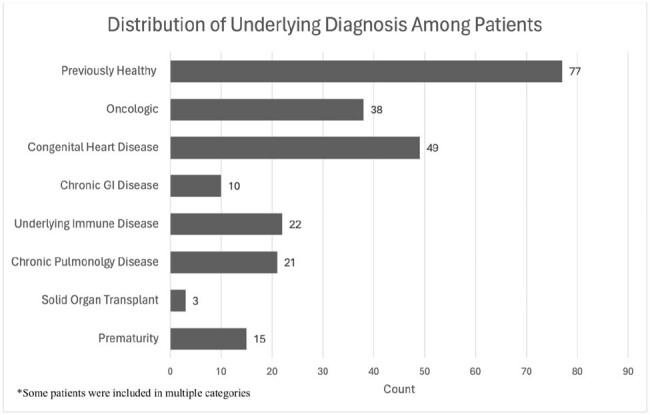
Distribution of Underlying Diagnosis in Sample

**Results:**

Evaluation of underlying diagnoses and the clinical usefulness of the Karius test revealed a significant association only for patients with an oncologic diagnosis (p=0.038) (Fig 3).

**Figure 3:**
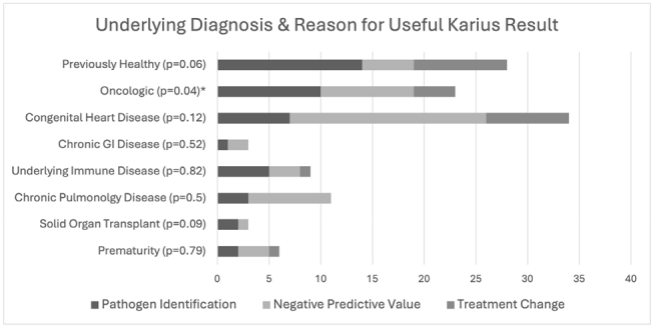
Underlying Diagnosis and Reason Karius Result Deemed Clinically Useful

**Conclusion:**

While Karius testing can be useful in specific patients regardless of the underlying diagnosis, this analysis found the most significant utility among patients with an oncology diagnosis. Further investigation through larger sample sizes and prospective designs is essential to validate and extend these findings, shedding light on cost-effectiveness and patient outcomes.

**Disclosures:**

All Authors: No reported disclosures

